# Chronic Unpredictable Mild Stress Model of Depression: Possible Sources of Poor Reproducibility and Latent Variables

**DOI:** 10.3390/biology11111621

**Published:** 2022-11-06

**Authors:** Dmitrii D. Markov, Ekaterina V. Novosadova

**Affiliations:** Institute of Molecular Genetics of National Research Centre “Kurchatov Institute”, Kurchatova Sq.2, 123182 Moscow, Russia

**Keywords:** chronic unpredictable mild stress, sleep deprivation, pain, social stress, handling, habituation, physical stressors, psychological stressors, vocalization

## Abstract

**Simple Summary:**

Scientists use animal models to study the mechanisms of human diseases and find new drugs to treat them. Chronic Unpredictable Mild Stress is the best animal model of depression. However, this animal model is very sensitive to even the slightest changes in design. Researchers often have difficulty reproducing the main behavioral and physiological effects of Chronic Unpredictable Mild Stress. Animal behavior is influenced by a wide variety of factors. The better we standardize the Chronic Unpredictable Mild Stress procedure and the more factors we can control; the more reproducible results will be obtained by scientists from different laboratories who are engaged in the study of depression. In this review, we have attempted to identify possible sources of poor reproducibility of this model and have made a number of recommendations that can improve the model by making it more reliable.

**Abstract:**

Major depressive disorder (MDD) is one of the most common mood disorders worldwide. A lack of understanding of the exact neurobiological mechanisms of depression complicates the search for new effective drugs. Animal models are an important tool in the search for new approaches to the treatment of this disorder. All animal models of depression have certain advantages and disadvantages. We often hear that the main drawback of the chronic unpredictable mild stress (CUMS) model of depression is its poor reproducibility, but rarely does anyone try to find the real causes and sources of such poor reproducibility. Analyzing the articles available in the PubMed database, we tried to identify the factors that may be the sources of the poor reproducibility of CUMS. Among such factors, there may be chronic sleep deprivation, painful stressors, social stress, the difference in sex and age of animals, different stress susceptibility of different animal strains, handling quality, habituation to stressful factors, various combinations of physical and psychological stressors in the CUMS protocol, the influence of olfactory and auditory stimuli on animals, as well as the possible influence of various other factors that are rarely taken into account by researchers. We assume that careful inspection of these factors will increase the reproducibility of the CUMS model between laboratories and allow to make the interpretation of the obtained results and their comparison between laboratories to be more adequate.

## 1. Introduction

While there are certain fundamental biological similarities between humans and rodents, humans are not just big rats. Rats should not be anthropomorphized. Results extrapolation from animal experiments to humans should be performed with caution. Can we really understand if a rat is depressed? How does a rat experience pleasure? What does a rat really feel? So far, we cannot answer these questions. However, experimental models are an important tool in the study of fundamental biological processes and mechanisms. Animal models are especially important in neuroscience, where it is still difficult to imagine the widespread use of invasive methods to study the human brain.

Modeling depression in animals is challenging for researchers. First, depression is a highly heterogeneous disorder. At least 5 of 9 symptoms must be present for a diagnosis, including at least one of the two core symptoms. It follows that 227 combinations of symptoms are possible. This leads to such diversity that two patients diagnosed with depression may have only one symptom in common [1]. Secondly, a number of symptoms that a depressed patient has cannot be modeled and evaluated in animals (feelings of guilt, worthlessness, feelings of sadness, suicidal thoughts, etc.). Third, the biological mechanisms underlying the development of depression remain poorly understood. However, now it is accepted that stress increases the risk of depression in humans, and therefore stress models are often applied to animals.

CUMS is one of the most common and frequently used models of depression, and it is the best attempt to simulate the human state in animals. However, the difficulty of reproducing this model by different laboratories and the low reproducibility of the results indicate the need to improve CUMS protocol.

Chronic stress was proposed as a model of depression in 1982 by R. Katz [2]. However, the author used some rather strong stressful factors. In 1987, P. Willner adopted the methodology of R. Katz by introducing more naturalistic stressful factors and proposed a chronic unpredictable mild stress model [3]. The CUMS model implies the exposure of animals to different stress factors in an unpredictable manner. CUMS leads to disruption of homeostasis, causing somatic, physiological, neurobiological, biochemical, and behavioral disturbances. One of the main consequences of CUMS is anhedonia, the inability to experience pleasure, a condition that is often assessed in rodents by their preference for sucrose solution. Since its introduction, the CUMS model has become very popular and has been actively used by laboratories around the world. However, some researchers began to point out the model’s weaknesses in the 90s, questioning the predictive, construct, and face validity of the model [4]. At the same time, despite active criticism of the CUMS model, P. Willner continued to insist on its reliability [5], and recent meta-analysis indicated the reliability of CUMS as an animal model of depression [6]. However, the results of this work may be compromised by a large amount of unpublished data. CUMS does not show equal results among different laboratories, and it is difficult to predict how many laboratories have tried to replicate the effects of CUMS but have not achieved the expected result and have not published these data. P. Willner himself attempted to interview 170 laboratories that have ever published articles using CUMS. The feedback was received from 71 respondents, which is 42% [7]. In addition, 58% of recipients prefer not to answer the question about the reliability of the CUMS model. In addition, we do not know the true reason for this, but we can assume that the authors experienced some problems with the reproducibility of the main effects of CUMS. Moreover, it is known that the statistical power of studies in neurosciences is very low. The consequences of this include overestimate of effect size and low reproducibility of results [8].

As already mentioned, not all laboratories are able to reproduce the main effects of CUMS; especially since this refers to a decrease in the sucrose preference in all stressed animals. Some researchers have come to revise the CUMS model of depression as it is now obvious that not all animals exposed to CUMS develop anhedonia. Chronic stress causes various physiological changes that are not associated with depressive-like behavior and anhedonia [9]. One of the most characteristic signs of the effectiveness of chronic stress is the reduction in body weight. However, a simultaneous decrease in consumption and preference for sweet solutions may not occur [10,11,12]. Of course, there is no doubt that chronic stress has a significant effect on the physiological state of all animals without exception, but only some of them reduce the preference for sweet solutions, while an increase in anxiety, changes in locomotor activity, and a decrease in body weight are a consequence of chronic stress which is not associated with anhedonia [13]. For those animals which do not show disturbances in hedonic behavior, Strekalova et al. suggest using as an internal control to assess the effects of stress which are not related to depressive-like behavior and anhedonia [14].

Strekalova et al. proposes to revise the CUMS model in order to improve it and increase its reliability and reproducibility. For example, they suggest using ethologically relevant stressors with an emotional component (instead of physical stressors) to omit food and water deprivation as well as poorly tunable stressors, such as continuous lighting, wet bedding, or social defeat. The authors also point out that it is necessary to pay attention to the factors that may impact the outcome of the sucrose preference test (stressors during the test, sugar concentration, diet, neophobia, social status of rodents, sensitization to reward experiences during repeated or prolonged exposure to palatable solutions, strain and inter-individual variabilities in liquid and sucrose intake, circadian rhythms and inter-batch variability) [15].

We have already discussed in our previous review the possible influence of an inadequately selected concentration of sucrose solution, water and food deprivation, stress-resilience, and stress-susceptibility of the animals on CUMS reproducibility [16]. We have proposed several recommendations to improve the reproducibility of the model, such as the determination of the minimal (or optimal) concentration of the sucrose solution before the CUMS, determination of the basal level of sucrose consumption/preference before the CUMS in order to identify animals with low, high and/or unstable sucrose consumption, exclusion of water and food deprivation from the protocol and segregation of animals into groups with a high and low responses to stressors (stress-susceptible and stress-resilient animals). In this review, we would like to consider other factors that may affect the reproducibility, such as chronic sleep deprivation, painful stressors, social stress, the difference in sex and age of animals, different stress susceptibility of different animal strains, handling quality, habituation to stressful factors, various combinations of physical and psychological stressors in the CUMS protocol, the influence of olfactory and auditory stimuli on animals (Table 1).

## 2. Chronic Sleep Deprivation

Commonly CUMS protocol is carried out during daylight, when rodents, such as nocturnal animals, are usually asleep. Due to the regular exposure of animals to stressful factors during daylight, which is an inactive period for them, they experience chronic sleep deprivation, which itself is an additional stressful factor and certainly affects the physiological state of animals, but most researchers do not usually take into account this fact. Sleep deprivation leads to the activation of the main neuroendocrine systems of the organism: the sympathoadrenal and hypothalamic-pituitary-adrenal axis (HPAA). In the long run, it may affect the reactivity of these systems to the stressors [17]. For example, it was shown that the adrenocorticotropic hormone (ACTH) response to restraint stress was significantly reduced after 48 h of total sleep deprivation or after 8 days of sleep restriction. It means that sleep deprivation affects the HPAA response to subsequent stressors [18]. This indicates that chronic sleep deprivation, which often occurs in CUMS procedures, will influence the endocrine response to a novel stressor used in CUMS protocol. The effect of rapid eye movement (REM) sleep deprivation on HPAA activation [19] was confirmed by an increase in the level of ACTH and corticosterone in the blood [20,21], an increase in corticotropin-releasing hormone (CRH) expression [22] and an increase in adrenal weight [23]. Sleep deprivation in humans also leads to disturbances in HPAA functioning [24].

Moreover, REM sleep deprivation causes a decrease in body weight [23]. It is well known that a decrease in the body weight of animals is observed in the vast majority of studies where the protocol of CUMS was used, and this is one of the most reproducible effects. It was shown that chronic sleep restriction in rats leads to a significant attenuation of weight gain [25,26], often accompanied by hyperphagia [27,28]. In addition, it can take quite a lot of time to restore body weight to a normal level. After 10–12 days of REM sleep deprivation, it takes about 5 days for 2-month-old rats to restore their body weight, about 10 days for 6-month-old rats, and more than 35 days for 12-month-old animals [29]. When taking into account these data, it cannot be ruled out that weight loss during CUMS may be the result of chronic sleep deprivation.

It was shown that chronic sleep disruption induces depression-like behavior in both adolescent male and female mice [30]. However, at the same time, it is well known that sleep deprivation has an antidepressant effect in patients with depression [31,32,33], alleviating depressive symptoms in 60% of them [34,35]. More research is needed to explain these inconsistencies between animal and human studies.

In humans, sleep deprivation leads to increased consumption and cravings for palatable food [36,37,38,39]. After sleep curtailment, a significant increase in the preferred concentration for sucrose was observed in healthy participants [40]. Sweeter versions of the oat products were liked more after sleep curtailment [41]. Participants in the shorter sleep group prefer higher concentrations of sucrose solution than people in the longer sleep group [42]. Habitual long-sleepers prefer higher concentrations of sucrose solution after sleep curtailment [43]. However, there is conflicting evidence indicating the absence of any changes in the perception of sweet taste (solutions with varying sucrose concentrations) after total sleep deprivation [44].

REM sleep deprivation also leads to an increase in sucrose consumption [45] and other sweet foods by animals [46] but paradoxically causes a decrease in the motivation for food reward [47] and does not lead to changes in response rate or threshold for intracranial self-stimulation [48,49]. This fact indicates the dissociation between the consumption of sweet solutions and intracranial self-stimulation.

On the one hand, we expect that CUMS should lead to the development of anhedonia, which is expressed in a decrease in sucrose consumption/preference. On the other hand, animals are often subjected to sleep deprivation during the CUMS procedure, which in turn leads to an increase in sucrose consumption/preference. Thus, during CUMS, two oppositely directed processes simultaneously occur, competing with each other. Under such conditions, it becomes difficult to predict the final effect. In this regard, it is interesting to note the work of Jiang Y. and Zhu J., where it was shown that CUMS leads to a decrease in sucrose consumption. However, animals that were sleep deprived after CUMS, in contrast, increased their sucrose consumption [50].

It is also necessary to pay attention to the time of day when CUMS protocol is implemented. Rats and mice are nocturnal animals. However, in scientific laboratories, experiments with animals are carried out, as a rule, during the working day (light phase). It means that the active period of the experimenter corresponds to the rest period of the laboratory animal. Testing animals in their inactive phase can significantly affect their behavior and physiological parameters, causing stress, leading to loss of motivation, etc. [51]. Thus, for example, it was shown that animals are more vulnerable to a stressor that occurs during their rest phase. Uncontrollable tailshocks in the inactive phase elicited behavioral changes in rats. In contrast, rats that underwent uncontrollable tailshocks during the dark (active) phase were buffered against stress-induced changes [52]. Animals are less stressed when they are tested (forced swim test (FST)) during the active (dark) phase, as evidenced by a decrease in escape-oriented activity in FST and by a less significant increase in corticosterone level [53].

The same is true for the CUMS. It is worth noting that CUMS is effective only if the protocol is implemented in the daytime (inactive period for rats) and not in a dark phase of the diurnal cycle. When exposure to CUMS occurred during the light phase of the day cycle, rats displayed signs of depressive and anxiety-related behaviors (delayed body weight gain, reduction in sucrose solution consumption, increased anxiety in the elevated plus maze (EPM), disturbances in the pattern of corticosterone secretion) [54]. The time of day when sucrose consumption/preference is assessed is also very important. The main consumption of sucrose solution by mice [55] and rats [56] occurs during the active period (night). The consumption of a 1% sucrose solution by rats is almost three times higher during the dark phase as compared to the light phase [57]. Moreover, the motivation for food reward in mice is higher during the nighttime [58]. This means that the best time to assess sucrose consumption/preference is the night period when the animals are in their active state. This is confirmed by the fact that CUMS leads to a decrease in sucrose intake and sucrose preference in animals tested at the dark phase but not in animals tested during the light phase [59].

Due to the fact that many stressful factors, which are components of CUMS, are presented to animals in the light phase when they should sleep, they experience chronic sleep deprivation. In turn, it is obvious that sleep deprivation has a significant impact on HPAA functioning, body weight, and consumption of sweet solutions. Depending on the duration of the whole protocol of CUMS and the duration of individual stressors, the degree of sleep deprivation can vary significantly between laboratories, causing poor reproducibility of the model. The effectiveness of CUMS will depend on the time of day when stressors are applied. The level of sucrose consumption/preference by the animals will depend on the duration of the sucrose preference test and the time of day.

## 3. Pain and Depressive-like Behavior

In 1980, Le Magnen J. et al., studying the effects of naloxone on the consumption of sweet solutions, suggested that the reward and pain modulating systems form a biochemical continuum in which the opioid system plays a central role [60]. Currently, there is evidence of common neurobiological mechanisms (brain regions, neurotransmitters, neuropeptides) of painful and pleasant sensations [61,62]. It is also suggested that there are common molecular mechanisms underlying chronic pain and depression comorbidity [63]. In this regard, it is noteworthy that the comorbidity of depression and chronic pain is observed in 80% of patients [64], and the prevalence of major depression in patients with chronic low back pain is 3–4 times greater than that reported in the general population [65]. Chronic pain in rats induces depressive behavior [66], and chronic neuropathy causes a decrease in sucrose consumption [45]. It is also known that in animals, chronic pain induces impairment in motivational reward behavior [67,68].

It cannot be ruled out that the pain experienced by animals during the CUMS can have a significant impact on their consumption of sweet solutions. Researchers led by Catherine Belzung, for example, recommend avoiding the use of severe stressors such as water and food deprivation and painful stressors. Firstly, they do not reflect the etiology of depression in humans, and secondly, they have a generalized effect on the physical state of the animal, which may interfere with the measures of the CUMS-induced effects [69]. For example, Harris R et al. used saline injection as one of the stressful factors in the CUMS protocol [10]. Injections are often employed in CUMS to test compounds with antidepressant properties. At the same time, it was shown that chronic intraperitoneal injections have a negative influence on animals, and the procedure itself is stressful [70,71]. It was shown that acute intraperitoneal saline injection leads to an increase in corticosterone and HPAA activation [72]. However, the situation with mice is not so straightforward. Acute intraperitoneal administration of saline to DBA/2J mice and C57BL/6J mice leads to the activation of various brain regions. C57BL/6J mice, unlike DBA/2J mice, successfully adapt to subsequent injections [73]. It was also shown that daily intraperitoneal injections of saline for 7 [74] or 30 days had no adverse effects on the behavior and physiological state of mice [75]. In contrast, Du Preez A. demonstrated that chronic 6-week stress (repeated injections) in adult male mice resulted in an anxiety-like phenotype, decreased systemic inflammation, and promoted HPAA hyperactivity [76]. Different laboratories use different injection techniques and have different injection schedules. In each specific laboratory, injections may be more or less painful for animals, and the degree of adaptation of animals to injections may also be different. In animal models of depression, experimental animals are usually injected with an antidepressant drug, and control animals are injected with saline. Since the injections themselves can be painful for animals and are a strong stressor, in order to control the effect of injections on the physical state of animals, we recommend having a group of intact animals that will receive no injection at all.

Not only the injections themselves but also the manipulations with the animal during the procedure (grasping, lifting into the air, turning over) may be considered as a series of aversive stimuli [77]. It is likely that the physical restraint of the animal (restraining the animal using a firm grip around the neck and shoulder or using a scruffing technique) during injections has an even greater influence on the animal than the injections themselves [78,79]. It is appropriate to mention here the study of Aydin C., in which it was shown that chronic intraperitoneal injections of saline significantly increased the time spent immobile in the forced swim test in high responders and significantly decreased the same measure in low responders. This work, firstly, indicates that chronic injections have a significant effect on depressive-like behavior and secondly, indicates that the behavior is largely determined by the emotional status of the animal (by individual differences in stress reactivity) [80].

Taking into account the possible effect of painful stimuli (for example, the intraperitoneal administration of antidepressants) on depressive-like behavior of animals, even minor pain should be avoided when animals are exposed to CUMS. Various methods of physical restraint, as well as some stressful factors (injections, tail pinch, electroshock, immobilization, restraint stress), can cause physical pain. The set of such factors that can cause pain in each laboratory will be different and may contribute to poor reproducibility of the CUMS between laboratories.

## 4. Social Stress

The majority of the stressful factors that influence human well-being is associated with social interactions. Among such factors, for example, are the death of a spouse, divorce, estrangement, imprisonment, death of a close family member or friend, family quarrels, the child’s leaving home, and social conflicts [81]. People with low-quality of social relationships have a two times higher risk of developing depression than people with high-quality of social relationships [82]. Rodents are also social animals, characterized by a hierarchical structure (subordination-domination system). It is well known that animals communicate with each other using tactile, olfactory, visual, and sound signals, the presence or absence of such signals has a significant impact on the functioning of the neuroendocrine systems of the organism. The consequences of exposure to a stressful factor largely depend on the social environment of a particular animal at the time of exposure to such a stressor. The presence of a conspecific can be both a stressful factor and a calming one, and this is determined by the specific situation. The effects of the stressful factors on the animal are less significant when the animals are group housed, an effect called social buffering [83]. Individually housed (isolated) rats, on the contrary, are characterized by “isolation syndrome” [84].

Social isolation, crowding, social instability, and social defeat are the commonly utilized stressful factors in CUMS and can affect the physiological state and behavior of the animal. Both crowding and isolation lead to increased levels of corticosterone, atrophy of the thymus gland, and adrenal hypertrophy [85]. Chronic social stress (resident-intruder paradigm) leads to the development of anhedonia, the suppression of locomotor and exploratory activity, weight loss, and an increase in adrenal weight [86]. Group housing, especially with frequent changes of cage mates, can also be stressful for animals [87].

Isolation occurs during the sucrose preference test, where individual housing is a prerequisite. It is also known that individual housing is stressful for animals [88]. However, the effects of social isolation on depressive-like behavior and other physiological parameters of the animal are controversial. For example, social isolation for over 3 weeks does not lead to the development of depressive-like behavior in rats [89], does not affect anxiety-like behaviors in rats, but leads to an increase in the levels of IL-6, TNF-alpha, and TLRs in the hippocampus [90] and to an increase in food intake [91]. At the same time, the individually housed mice show increased immobility time in the forced swim test, increased body weight, and reduced levels of corticosterone in comparison to group-housed animals [92]. Housing conditions (single and paired housing) can have a significant influence on the dynamics of animals’ weight gain and their sucrose preference. The lack of social contact in the single-housing condition makes animals more susceptible to stress [93]. Animals kept in isolation consume less saccharin solution upon the first presentation than animals kept in groups [94]. Juvenile isolation during 4–5 weeks decreases the motivational properties of sucrose-drinking [95].

It is also important to consider the hierarchical status of the animal in the experimental designs. Animals from different cages but with the same dominance rank have more similar phenotypic traits than cage-mates with different ranks [96]. As mentioned above, rodents form a hierarchical structure in their community. Subordinates are characterized by depressive-like symptoms, and show an increased level of corticosterone, lower body weight, reduced locomotor and social activity, and increased adrenal weight [97,98]. In human society, people with a subordinate status are also characterized by depressive symptoms, a sense of inferiority, and low self-esteem [99,100]. At the same time, it is assumed that dominant individuals are more vulnerable to stress exposure and have a high risk of developing depressive-like behavior than subordinates [101]. Dominant individuals are also more susceptible to chronic social defeat stress than subordinate mice [102].

It is obvious that social stress has a significant influence on the development of depressive-like behavior, and the use of social stress as an independent animal model of depression is the best evidence of this. Different laboratories can use various types of social stress (isolation, crowding, resident-intruder) and their various combinations. The duration of social stress can vary greatly, and the density of animals in both home cages and during crowding can also vary significantly. All of this can serve as reasons for the poor reproducibility of the CUMS between different laboratories.

## 5. Animal Strain

Strain, sex, supplier, and individual variability are the main sources of variability in the modeling of affective disorders and in their treatment [103]. Experiments performed by Catherine Belzung et al. demonstrate a strong influence of genetic background and biological sex on the susceptibility to CUMS [104]. Attention should be paid to the strain of animals, both when testing and when comparing results with data obtained by other laboratories, because animals of different strains differ in their susceptibility to stress. For example, CUMS induces a significant reduction in sucrose preference in both rat strains (Wistar and Sprague–Dawley), but Wistar rats show a more pronounced and stable decrease in sucrose preference [105]. The intake of 1% sucrose solution was significantly reduced in PVG-hooded rats exposed to CUMS but not in Wistar rats [106]. When comparing Lewis, Fischer (F344), and Sprague–Dawley rats, Fischer (F344) rats are the most sensitive to CUMS, showing a significant reduction in sucrose consumption, while Sprague–Dawley rats do not exhibit depressive behavior [107]. Different strains of mice have different susceptibilities to CUMS, as well as different responses to antidepressants. BALB/c and C57BL/6 mice are the most susceptible to CUMS and the most responsive to antidepressant treatment. C3H and CBA mice are the least susceptible to the effects of CUMS and do not respond to imipramine treatment [108]. It was also shown that BALB/c mice are a sensitive strain to the effects of CUMS in contrast to Swiss mice, and this is confirmed by the deterioration of the coat observed in BALB/c mice [109]. Inbred C57BL/6 and outbred ICR mice were similarly responsive to CUMS in the sucrose intake test and open field test. However, the two strains showed quite different responses in FST and novelty-suppressed feeding (NSF) test after 3 weeks of CUMS. Only C57BL/6 mice displayed depression- and anxiety-like behavioral effects in response to CUMS in FST and NSF tests [110]. Different strains of mice also have different susceptibilities to the subchronic version of CUMS. Subchronic CUMS induced deterioration of the fur state in FVB/NA, BALB/c, DBA/2, 129/Sv, C3H/He, and BA strains but not in C57/BL6 and CBA mice. It is interesting to note that at the same time, sucrose preference failed to decrease in most of the strains after CUMS except BA mice [111]. Different mice strains also have different sensitivity to antidepressants. For example, fluoxetine has an antidepressant effect in BALB/c mice and a prodepressant effect in C57BL6/J mice [112]. The different strains of mice exposed to CUMS are also characterized by variability in sucrose consumption [113]. Different sensitivity to the CUMS is observed even in animals of the same strain but from different suppliers. Wistar rats from Charles River show increased anxiety and reduced sucrose consumption in comparison to animals from Janvier [114]. This is probably due to the fact that different rat strains differ significantly in the HPAA activity [115]. Similar differences in the HPAA functioning were observed even in rats of the same strain but from different vendors [116]. It is necessary to take into account possible differences in the stress response of animals of different strains or animals of the same strain, but from different vendors, during the CUMS or when comparing your results with data from other laboratories.

## 6. Handling

The response of the organism to the action of nonspecific (not taken into consideration by the experimenter) stress factors depend on the degree of familiarity with the hands of the experimenter and the main manipulations. It is very important to carry out handling procedures by the same person, and if repeatedly handled rats are further handled by a different person, a dishabituation occurs immediately [117]. The point is that regular handling causes adaptive changes in the sympathoadrenal and hypothalamic-pituitary-adrenal axis. For example, repeated handling for 8 days reduces corticosterone level in rats [118]. Early postnatal handling leads to attenuated neuroendocrine stress response to a stressful situation in later life [119]. On the contrary, an increased level of corticosterone in mice indicates that mice do not habituate to handling over a 1-week period. Moreover, daily handling causes a ∼25% reduction in resting time [120]. Different researchers carry out handling in different ways, which means that the effect on the duration of the resting period of animals can be different. Different methods of handling (rough and gentle) can have diametrically opposite effects on animals, in particular on the activation of various brain regions [121,122]. The method of physical restraint during handling (scruff, encircling, plastic cone, lifting, and holding by the tail on the arm) is also important [123]. For example, tail handling has negative effects on mice behavior and causes a greater decrease in sucrose consumption in comparison to tunnel-handled mice [124]. Laboratory mice picked up by tail also exhibit strong aversion and increased anxiety [125]. On the contrary, gentle handling has been shown to reduce depressive-like behavior in mice, which is proved by reduced immobility in the forced swim test compared to mice that were aggressively handled [126].

In most cases, researchers do not provide a detailed description of the handling methods. At the same time, the importance of tactile contact between the experimenter and the experimental animal should not be underestimated. Touching animals, holding them, or physical restraint are stressful manipulations. It is obvious that before the beginning of the CUMS implementation, animals in different laboratories can be of varying degrees of familiarity with the experimenter. The handling method and its duration can influence the physiological state of animals. Some animals will be relatively calm, being in the hands of a researcher. For others, it will be a powerful stressful factor. The difference in the quality of handling in different laboratories may be the reason for the poor reproducibility of the CUMS model.

## 7. Habituation/Desensitization

In 1943, Harris J. attempted to summarize information about the process of habituation of organisms, implying by this term a decrease in the magnitude of the response to a specific stimulus with repeated exposure to that stimulus [127]. In 1966, Thompson R. and Spencer W. formulated the key features of the habituation to repeated exposure to the stimulus [128], which were revised with additions in 2009 [129]. At present, some researchers consider that declines in HPAA response to repeated stress are response habituation in a sense defined by Thompson and Spencer, with the remark that HPAA activity habituates to predominantly processive stressors and not to systemic stressors [130]. However, other authors consider that adaptation of the HPAA to daily repeated homotypic stress does not follow the rules of habituation. In particular, contrary to the main criteria proposed by Thompson R. and Spencer W. to severe stressors, adaptation develops faster and stronger (even with a single exposure to severe stressors) [131]. Long-term HPAA desensitization occurs only in response to the same (homotypic) stressor, while in response to novel (heterotypic) stressor, enhanced activation of HPAA is observed [132]. For example, enhanced HPAA response, expressed in an increase in the level of ACTH and corticosterone, occurred after exposure to the novel stressor (restraint) in rats previously exposed to chronic cold stress [133] and repeated social stress [134]. However, it is not always the case. For instance, ACTH and corticosterone responses to a novel environment were not affected by prior exposure to audiogenic or restraint stress [135].

It is interesting to note that two processes (habituation and sensitization) occur simultaneously and independently. Repeated ferret odor exposure for two weeks leads to habituation, which is expressed in a decrease in the level of ACTH, corticosterone, and expression of *c-fos* mRNA in the hypothalamus. If animals are subjected to a novel stressor (restraint stress) after two weeks of the same stressor (ferret odor), then a sensitization of the response is observed, which is expressed in increased levels of ACTH, corticosterone, and *c-fos* mRNA expression in the hypothalamus in such animals, compared with animals that were subjected to the restraint stress for the first time [136].

The adaptation of the sympathoadrenal system is also of interest. The plasma catecholamine response to restraint stress on Day 28 was significantly reduced in animals exposed to chronic homotypic stress (restraint stress, high predictability) and chronic variable stress (restraint, cold swim, or intermittent footshock, low predictability) compared to first-time stressed controls. These data indicate the development of adaptation of the sympathoadrenal system to repeated exposure to the same stressor and show that the predictability of the stress exposure does not affect the development of adaptation [137].

The cardiovascular system also habituates to repeated exposure to the stressor (restraint stress). Cardiovascular responses decrease upon repeated exposure depending on the length, frequency, and the number of exposures [138].

It is assumed that habituation to the exposure to the repeated stressor occurs due to nonassociative learning [139]. It cannot be ruled out that such an effect of HPAA desensitization is associated with the process of memory formation and has evolutionary/adaptive value. The physiological and behavioral changes in response to a novel emotional stressor constitute an anticipatory response aiming to better cope with stress. Emotional stress can pose a real threat to the organism and may not pose any danger at all. Nevertheless, the organism should be prepared at any moment that a potential threat can become real with negative consequences for the organism. For example, when facing a predator, the victim should be ready to escape in order not to be killed or wounded, and in this case, it is necessary for all the systems of the organism (muscular, cardiovascular, respiratory, etc.) to be prepared for this scenario. However, such a response (maximal activation of all systems) is energy-consuming and inappropriate if the stimulus does not pose a real threat to survival. Repeated exposure to the same stressful factor initiates two processes: one of them is directed to the activation of the neuroendocrine system, and the second-to modulation of activation based on previous experience. The second process is associated with the processes of learning and memory formation. The final level of activation of the neuroendocrine system will be the result of the integration of two processes. If previous experience suggests a safe situation, then the activation of the neuroendocrine system will be inhibited [131].

As follows from the name, chronic mild stress is “unpredictable.” There is no doubt that the time of presentation of the stressful factors is indeed unpredictable for the animal. However, the unpredictability of the stressor to the animal after the first presentation of it is doubtful because even after the single presentation, the animal becomes familiar with this stressor. It has been shown, for example, that a single exposure to immobilization stress is sufficient to ensure a more rapid return to the baseline of the levels of ACTH and corticosterone upon subsequent exposure to the same stressor days later [140]. It does not matter when exactly the animal will be immobilized again, one week or four weeks after the first immobilization [141]. Such effect of HPAA desensitization, partially mediated by glucocorticoids [142], is associated with adaptive changes in the brain [143] and is observed in both Sprague-Dawley and Wistar rats [144].

One of the illustrative examples of the development of an adaptive response after the first presentation of a stressor (pretest) is the forced swim test, which is widely used as an independent stress factor and a test for assessing depressive-like behavior. A large number of researchers indicate that increased immobility in the FST on the second day (test) is associated with learning processes and adaptation [145,146,147,148,149,150].

It is believed that CUMS mirrors uncontrolled stress experienced by individuals and prevents habituation from stressing exposure. In the CUMS model, stressful factors are presented in a nominally unpredictable order. These factors are multimodal, and each of them is presented, as a rule, several times during the implementation of the protocol. It cannot be excluded that animals repeatedly exposed to the same stressful factors stop to perceive them as a real threat, realizing that after some time, they will be safely returned to their home cage. In this case, the effect of each stressful factor on the organism and their cumulative effect during CUMS will be determined by the complex interaction of habituation, desensitization, facilitation, learning, and memory formation.

Currently, there are very few works studying the overlapping effects of different stress factors during CUMS. Existing data indicate that repeated exposure to the same stressor is less stressful for the animal compared to its first presentation. In particular, if animals are divided into two groups and exposed to two variants of CUMS (with or without restraint stress), a reduced level of ACTH after exposure to novel restraint stress will be observed only in those animals that were subjected to CUMS with restraint stress included in the protocol. These data indicate that animals develop adaptation when repeatedly exposed to the same stressor during CUMS [151]. Exposure to a novel environment (unfamiliar stress) after CUMS with restraint stress included in protocol leads to an increased level of corticosterone. If animals are exposed to already familiar stress after CUMS (restraint stress), no changes in corticosterone levels are observed [152]. However, in a subsequent experiment, the authors were unable to reproduce the results. They found no change in corticosterone levels after exposure to both already familiar (restraint stress) and unfamiliar stressors (novel environment) [153]. In addition, on days 4 and 7, but not 16 h after the cessation of CUMS, a decrease in the HPAA activation is observed even when animals are exposed to new (unfamiliar) stressors such as novel environment and restraint stress [154].

The disappearance of effects over time during CUMS can serve as evidence in favor of habituation and confirms that animals become accustomed to stress factors. Researchers differ in opinion when the weakening of the effects of CUMS occurs. A decrease in the sucrose preference can be observed after the first week of CUMS and may disappear later, which may indicate an adaptation of animals to stressful factors [155]. Grønli J. observed the highest decrease in sucrose consumption after two weeks of CUMS, and later this effect became less pronounced, as evidenced by an increase in consumption [156]. Pothion S. showed that a decrease in the sucrose consumption in mice was observed during the first four weeks of CUMS, then the effect of stress became less significant, and the sucrose consumption was restored or even tended to increase [113]. The presence of adaptive changes to the long-term CUMS procedure in mice is also supported by the fact that depressive-like behavior (in the splash test, FST, and tail suspension test (TST)) was more pronounced in mice stressed for 18 days than for 36 days of CUMS. The same trend was observed for plasma corticosterone levels but not for the dynamics of weight gain. A decrease in weight gain was observed as the duration of the CUMS procedure increased. It means that depressive-like behavior is not a result of weight loss but rather develops independently [157].

The time interval between the stressors during the CUMS also plays an important role. Attenuation of body weight gain and decrease in sucrose preference are more significant if stressors are clustered (stressors that occur within 1-hour intervals) but not when they are dispersed during the day [158]. In addition, the duration of exposure to stressful factors used in CUMS can vary significantly (from several minutes to several hours).

Of course, these phenomena require additional research, especially in the case of CUMS, but in order for stress to be really “unpredictable,” in our opinion, reusing the same stressors should be avoided, even if they are employed in an unpredictable manner. At the same time, a limited set of stressors, means that the longer duration of the CUMS protocol and the fewer stressors in its design, the more likely the animals will adapt to these stressors and the more predictable (familiar) the protocol will be for these animals. Differences in the adaptation of animals to stressful factors in different laboratories may be the reason for poor reproducibility.

## 8. Age

Using the CUMS, researchers often indicate the body weight of animals, implying that body weight indicates age. However, it is well known that body weight only approximately reflects the age of the animal and may not correspond to it. In addition, such definitions as a juvenile, adolescent, and adult are often found in articles and also characterize the stage of postnatal development but do not indicate the exact age. The actual age of animals that meet the definition of adolescent or adult may vary significantly in different laboratories. Such definitions, without indicating the exact age, make it difficult to compare the results between laboratories and complicate their extrapolation (if it is appropriate to speak of it) to humans.

The development of rodents can be divided into the following stages: prenatal period, early postnatal period, adolescence (early, intermediate and late periods), adulthood, and post-reproductive period. It is known that on the 21st day of postnatal development of rats, time spent suckling begins to decline, and there is a simultaneous increase in the consumption of solid food. By the 34th day of postnatal development, as a rule, the young no longer suckle, and weaning is essentially complete. Animals become sexually mature by an average of 42 days of postnatal development, reaching adulthood at 63 days of postnatal life [159]). Sexual maturation in female rats occurs somewhat earlier (32–34 days) than in males (45–48 days) [160].

Regarding the comparison of the age of the rat with the age of a human, we can say the following: if we accept that the life span of a human is, on average, 80 years and for a rat it is 3 years, then 13.7 days of a rat’s life are equivalent to one year of human life. However, it is worth noting that this is a rather crude and formal approximation [161]. In adulthood, 1 month of rat life is approximately equivalent to 2.5 years of human life. For example, an animal aged 12 months will correspond to a human aged 30 years and an animal aged 24 months to a human aged 60 years [162].

For experimental purposes, researchers mainly use young animals. At the same time, in human society, people aged 18–59 suffer from depression [163,164].

Animals of different ages differ in their susceptibility to stress. HPAA activation in response to acute and chronic stress varies depending on the stage of animal development [165]. There are few studies that simultaneously evaluate the effects of CUMS on animals of different ages; the available data indicate significant differences in stress susceptibility of animals of different ages. CUMS affects adolescent animals (40 days) and adult animals (60 days) in different ways, which is confirmed by differences in their behavior in EPM and FST, as well as by differences in the HPAA activation after restraint stress [166]. Anhedonia, decreased neurogenesis and a decrease in the level of BDNF in the hippocampus occur only in adult animals (60 days-old), while young animals (30 days-old) adapt to CUMS [167]. According to Herrera-Pérez J., only 35% of rats aged 3–5 months reduce the intake of sucrose solution during the CUMS, while at the age of 12–15 months, the number of anhedonic animals increases to 73% [168]. According to other researchers, anhedonia as a result of CUMS develops in both young (2 months old) and adult animals (15 months old), but only in the latter a decrease in the sucrose preference is observed on day eight after the cessation of CUMS, which indicates its greater impact on adult animals [169]. CUMS exposure resulted in decreased body weight and increased adrenal size in all animals regardless of age (early adolescence (35 days), late adolescence (50 days), adulthood (80 days)) but an increase in immobility time in the forced swim test is observed only in adult animals [170]. At the same time, there are directly opposite data when adrenal hypertrophy, thymic involution, and elevated level of glucocorticoids were observed only in adolescent rats (28 days) but not in adult animals (60 days), whereas reduction in body weight was observed only in adults [171]. Depressive-like behavior is observed in both adolescent mice (PND28) and adult animals (PND70) exposed to CUMS, but anxiety-like behavior increases only in adolescent mice [172].

Animals at various stages of their postnatal development may be more or less susceptible to stressors, and the development or absence of depressive-like behavior in animals exposed to CUMS will depend on their susceptibility. This means that the use of animals of different ages by different research groups can contribute to poor reproducibility of CUMS model and inadequate comparison of the results.

## 9. Sex

It is well known that stressful situations are much more likely to provoke the development of depression in women than in men [173]. Depression is almost two times more likely to develop in women than in men [174,175,176]. Perhaps the reason for the high prevalence of depression in women is the change in the level of reproductive steroids [177]. The molecular mechanisms of depression in men and women seem to have some differences. For example, it was shown that depression in women and men is associated with a change in the expression of various genes in the brain and the overlap between males and females in the transcriptional pattern is less than 10% [178]. In 1993, the US National Institutes of Health published the «National Institutes of Health Revitalization Act of 1993», which recommended increasing the representation of women in ongoing clinical trials. These recommendations helped to improve the situation in medicine but, unfortunately, did not significantly affect the representation of females in animal models used in neuroscience, despite repeated calls for the need to change the situation [179,180]. Currently, in such areas as neurobiology, pharmacology, endocrinology, and physiology, works that use only males continue to dominate. In particular, in neuroscience, the ratio of the number of articles in which males are used to the number of experimental works using individuals of the opposite sex is 5.5:1 [181]. Due to the fact that the situation does not change significantly, the US National Institutes of Health now strongly recommends not neglecting the use of females in animal models and even cell cultures [182]. The rejection of the use of females in neurobiological experiments is most often justified by the possible influence of hormonal fluctuations associated with the reproductive cycle of females on the measured parameters. However, the results of the meta-analysis do not confirm such concerns because no difference in the variability of data obtained in behavioral, electrophysiological, and neurochemical experiments using males and females was found [183,184].

It is well known that females and males perceive and respond differently to the same stimuli. There are sex differences in depressive-like and anxious behavior, learning and memory, levels of neurotransmitters and neurotrophic factors, neurogenesis, and synaptic plasticity [185]. Such sex-differences can be explained by gonadal hormones, chromosome complement, or both factors simultaneously [186]. It should be emphasized that the stress response and the HPAA functioning in females have distinctive features, which make them more susceptible to stressful situations [187,188]. Stress in females leads to more prominent activation of HPAA, which is confirmed by higher levels of corticosterone and ACTH [189].

Neurochemical, neuroendocrine, and behavioral differences between females and males were found in all major depression models and tests for evaluating depressive-like behavior [190,191,192]. It is well known that CUMS affects males and females differently. This is proven by the differences in the sucrose preference test, the processes of learning and memory formation, the forced swim test, their locomotor activity, neurochemical parameters, neurogenesis, and in HPAA and immune system functioning [193]. Female and male rats exposed to CUMS, differ in their hormonal profile, hypothalamic mRNA expression of stress-related molecules [194], and the level of different proteins in the hippocampus [195]. In general, it is believed that for female rats, the effects of CUMS are more adverse [196]. For instance, an increase in corticosterone levels occurs only in females but not in males [197]. Other researchers also point out that an increase in corticosterone levels occurs only in females exposed to CUMS, while a decrease in body weight and adrenal hypertrophy is observed only in males [198]. CUMS has a more pronounced negative effect on female rats, and this is confirmed not only by a more significant increase in serum corticosterone level but also by a more significant reduction in exploratory behavior in the open field and a more significant decrease in the sucrose preference [199]. However, the results of various research groups are quite contradictory. For instance, it was shown that CUMS leads to a decrease in sucrose consumption in both female and male rats. However, the most significant and stable decrease is observed only in males [200]. Despite the obvious differences between females and males, currently, male rodents are used in the CUMS model of depression much more frequently than females [201].

In mice, females are also more susceptible to CUMS compared to males. In particular, females exposed to CUMS have a slower rate of weight gain, a more pronounced decrease in sucrose preference, and a higher level of corticosterone in serum [202]. Female mice exhibit more pronounced despair-like behavior in the forced swim test compared to males [203], or depressive-like behavior (including reduced sucrose preference) develops exclusively in females [204]. CUMS affects male and female C57BL/6 mice differently. This is expressed in varying degrees of changes in serum levels of neurotransmitters (5-HT, norepinephrine) and hormones (androgen, estrogen, oxytocin, corticosterone) [205]. It was also shown that, in general, responses to chronic variable stress (CVS) were similar between males and females in C57BL/6J mice. In both sexes, CVS induced a significant decrease in body weight, adrenal gland weight was not significantly different between control and CVS groups, but in females, a reduction in thymus weight was observed [206]. It is interesting to note that males, intact females, and ovariectomized female mice exposed to CUMS significantly differ in the profile of mRNA expression in the hypothalamus [207]. Moreover, the development of anhedonia [208] and depressive-like behavior in the forced swim test after CUMS occurs only in intact females [209,210]. However, the results are also contradictory since other researchers reported the opposite results. For example, it has been shown that ovariectomy enhances anxiety and depressive-like behaviors in mice submitted to CUMS compared to control animals, and this means that the absence of ovarian hormones enhances the effects of CUMS [211]. Either way, these data confirm the important role of female gonadal hormones in the development of depressive-like behavior.

Assessing depressive-like behavior in females using the sucrose preference test, the possible effect of gonadal hormones on taste responsivity to sweet solutions should be taken into account. In 1967, Valenstein E. found that female rats consume significantly more glucose and saccharin solutions than males [212]. Female rats also show a higher breakpoint in progressive ratio schedules of reinforcement and consume more sucrose pellets [213]. Subsequently, it became clear that the taste responsivity to sweet solutions in female rats depends on the stage of the estrous cycle. In particular, females in the diestrus/proestrus (with a high level of estradiol) are characterized by an increase in the preference for a sucrose solution, and in the estrus/metestrus (with a low level of estradiol) by a decrease in the preference of a sucrose solution [214]. Estrogens seem to increase the taste threshold for sucrose [215], and probably because of this, female rats are less sensitive to the rewarding properties of lower concentrations of sucrose [216]. In women, the sucrose threshold also depends on the stage of the menstrual cycle [217].

Obviously, CUMS affects females and males in different ways. Therefore, it is not surprising that males and females exposed to CUMS may differ in physiological and behavioral parameters. The mechanisms of different susceptibility of females and males are currently not clear, but their study can shed light on the pathophysiology of depression and HPAA functioning and contribute to the development of new, more effective therapeutic drugs with antidepressant properties. Hence, it is necessary to increase the representation of females in clinical trials and animal models.

## 10. Type of Stressful Factors (Physical and Emotional Stressors)

Despite the fact that Selye H. claimed that the response to stress is nonspecific [218], this statement is questioned now. Each stressful factor has its own neurochemical and neuroendocrine profile. Nonspecificity most likely means that any stressful factor, regardless of its nature, causes the activation of HPAA and the sympathoadrenal system.

Currently, a clear classification of stressful factors does not exist. For example, Pacák K. and Palkovits M. distinguish four groups of stressors: physical, psychological, social, and stressors that challenge cardiovascular and metabolic homeostasis [219]. Sawchenko P. et al. divide stressors into systemic and neurogenic and suggest calling them interoceptive and exteroceptive, respectively [220]. The division into two categories was made in 1951 by Fortier C., who studied the mechanisms of ACTH secretion and distinguished neurotropic stress factors (sound, immobilization) and systemic (cold stress, adrenaline, and histamine administration) [221]. Most researchers follow such a classification, dividing all stressful factors into two categories: physical stressors (reactive, interoceptive, systemic, homeostatic) and neurogenic (processive, psychogenic, exteroceptive, psychological, emotional). Systemic stressors include food deprivation, heat, and cold stress, immune stress, hypoglycemic stress, and ether stress. Processive stressors include restraint stress, novel environment, electric shock, isolation stress, and predator’s odor. In fact, many stressful factors are difficult to attribute to one category because they are multimodal, with the predominance of one or another subcomponent. For example, restraint stress is predominantly psychological, cold stress is more likely metabolic, and forced swimming is both a psychological and metabolic stressor.

Stress-specific activation of HPAA and the sympathoadrenal system is expressed in significantly different plasma levels of ACTH, epinephrine, and norepinephrine during immobilization stress, intravenous insulin administration, cold stress, experimental hemorrhage, subcutaneous formaldehyde administration, subcutaneous saline administration. It is interesting to note that the greatest increase in norepinephrine and ACTH levels occurs when animals are exposed to immobilization stress [222,223]. Nadal R. showed that ACTH, corticosterone, and prolactin response to the forced swim and immobilization in male rats was much higher than for open-field and elevated platforms [224]. Other authors also demonstrate that in rats, immobilization stress causes more powerful activation of HPAA, which is confirmed by a higher level of ACTH and corticosterone than the odor of cat fur or urine [225,226].

It has been shown that restraint stress in animals causes the greatest increase in corticosterone levels, cold stress, and forced swimming also led to a significant increase, but to a lesser extent in comparison to restraint stress, isolation stress and handling cause the smallest increase in corticosterone levels [227]. The level of corticosterone is significantly higher during ether stress (systemic) than in a novel environment (processive) [228].

In addition to differences in the hormonal profile, stress factors belonging to different categories differ in their ability to activate different brain regions. It was shown that the processive stressor (open field) and systemic stressor (ether) induce the same expression of *c-fos* mRNA in the paraventricular nucleus of the hypothalamus, but the expression pattern in other brain regions varies significantly [228]. It was shown that footshock (neurogenic stressor) and intravenous administration of IL-1 (systemic stressor) also cause the same induction of *c-fos* mRNA in the paraventricular nucleus of the hypothalamus, but a completely different expression pattern is observed in other brain regions [220]. Despite the fact that hypothalamic neurons become activated by any type of stressor, the profile of gene expression in the paraventricular nucleus after physiological (intraperitoneal administration of lipopolysaccharide) and emotional stress (restraint stress) varies significantly [229]. Physical (hemorrhage, immune challenge) and psychological (noise, restraint) stressors also lead to the activation of different parts of the amygdala [230]. In humans, psychosocial and physiological stress also causes the activation/deactivation of different brain regions [231].

If stressors belong to the same category, this does not mean that they activate completely identical brain regions. Despite the fact that swim stress (active movement) and restraint stress (movement restriction) are both processive stressors and have an overlapping pattern of *c-fos* mRNA expression in the brain, each of them also leads to the activation of distinct brain regions [232]. Emotional stressors of different intensities (open field, predator odor, immobilization) have different effects on the level of ACTH and have their specific pattern of *c-fos* expression in the brain. The highest level of ACTH is observed during immobilization and the lowest in the novel environment (open field) [233].

To process incoming sensory information during the exposure of stressors belonging to different categories, activation of different brain regions is required. Stressful stimuli can pose a “real” or a “false” threat to the organism. Stressful factors representing a real threat cause true homeostasis disturbance (disturbances in cardiovascular and respiratory systems, pain, inflammatory response). Stressful factors are representing a “false” threat that causes HPAA activation in the absence of real physical damage to the organism (in the absence of real homeostasis disturbances). It means that activation occurs in response to the expectation of danger. Such a reaction can be associated with learning processes and rely on previous experience (the appearance of a predator) or be an innate predisposition (unfamiliar environment, fear of heights, and open spaces). In general, it is assumed that stressful factors that do not pose a real threat to physiological homeostasis (restraint stress, a novel environment-processive stressor) can be perceived by the organism as truly stressful (and then activate HPAA) or as “false” (and then inhibit excessive activation of HPAA). In order to attribute the processive stressor as real or false, the brain needs to compare the current stressful situation with previous experience. Sensory information about such stressors requires additional preliminary processing in the forebrain (prefrontal cortex, hippocampus, amygdala). Systemic stressors (ether stress, hypoxia) always pose a real threat to the physiological state and do not require additional interpretation by higher brain structures. Such stressors directly (or via brainstem catecholaminergic projections) activate the paraventricular nucleus of the hypothalamus [234,235].

The type of stress may indeed matter, as was shown by Du Preez A. in a systematic review. Physical stress usually results in depressive-like behavior, impaired social interaction, and decreased body weight, while increased anxiety- and depressive-like behavior, impaired social interaction, learning and memory, increased HPAA activity, peripheral inflammation, microglial activation, and decreased hippocampal neurogenesis are all hallmarks of psychosocial stress [236]. A comparison of the effects of 28-day chronic stress based on physical stressors (forced swim, restraint, loud noise, cold exposure, ether exposure) and chronic stress based on social stressors (isolation, novel environment, crowding, litter-shifting, subordination), indicates that unpredictable physical chronic stress has a more significant negative effect on animals than unpredictable chronic social stress. This is confirmed by a more significant increase in adrenal gland weight and a more significant decrease in thymus weight and body weight in animals exposed to chronic unpredictable physical stress [237]. Different models of depression (CUMS, learned helplessness, chronic restraint stress, social defeat) prevail, with stressors belonging to different categories (physical and psychological). It is interesting to note that exposure to stressors of different natures leads to different changes in the level of metabolites in the hippocampus [238].

Stress factors belonging to different types differ in their ability to induce depression-like behavior. For instance, different forms of chronic stress (repeated injection and social isolation) can differentially alter both behavioral and biological outcomes in adult male mice. Exposure to 6 weeks of repeated injection resulted in an anxiety-like phenotype, hypercortisolemia, and reduced inflammation. In contrast, exposure to 6 weeks of social isolation resulted in a depressive-like phenotype, hypocortisolemia, and increased inflammation. It is interesting to note that combining such different stressors does not have a synergistic, potentiating effect and does not induce depressive-like behavior [76]. Kavushansky A. also showed that psychophysical (electrical foot shock) and psychological (social defeat) stress have different effects on anxious and depressive-like animal behavior, corticosterone levels, and gene expression in the brain. In particular, depressive-like behavior is observed only in animals subjected to psychological stress [239].

The type of stressor can differently affect the preference for sweet solutions in animals. Physical stress (foot shock) induces a decrease in preference for saccharine and open field activity compared to control. Animals subjected to emotional stress (witness) show an increase in open-field activity and a slight increase in saccharine preference. This means that physical stress causes anhedonia, while emotional stress causes an increased sensitivity to reward [240].

Some stressors can be as effective in inducing anhedonia as chronic stress. For instance, control mice showed a greater preference for sucrose solution compared to mice subjected to predatory stress or CUMS. This means that chronic psychological stress is as effective as CUMS in inducing anhedonia in mice [241]. At the same time, increased immobility in the TST and FST and reduced sucrose preference were only observed in mice exposed to CUMS but not to chronic restraint stress [242].

Stressors of different modalities during CUMS can superimpose and overlap each other, activating different brain regions and having their own specific hormonal profile. The resulting effect will depend on the complex interaction of effects caused by different stressors. Various research groups use a diverse combination of stressors in CUMS, and this may be one of the reasons for the poor reproducibility of the model and the CUMS effects.

## 11. The Odor of a Stressed Conspecific and Vocalizations

Animals receive information about the environment using visual, auditory, and olfactory sensations. Animals witnessing how stressful factors affect their conspecifics may themselves experience emotional stress. For example, mice subjected to emotional stress (witnessing social defeat) have increased levels of serum corticosterone, reduced weight gain, and exhibit depression- and anxiety-like behaviors similar to animals really subjected to social defeat. It means that witnessing stressful events applied to conspecifics is a potent stressor for the animals [243]. Animals acquire information from a stressed conspecific also via olfaction [244]. Rats, exposed to stress, secrete a specific odor. So-called alarm pheromones were identified in mice [245] and rats [246]. It is well known that rats can reliably distinguish between the odors of stressed and unstressed conspecifics [247]. The odor of a predator or a stressed conspecific can serve as a signal of danger, can be stressful for recipient animals [248], and can affect the behavior of non-stressed conspecifics [249]. For example, the odor of a stressed conspecific increases the locomotor activity in rats, which probably indicates an increase in exploratory activity aimed at obtaining information about the environment and the source of the odor [250]. Urine from stressed rats (electric footshocks) increases open-field locomotor activity and immobility in receptor rats forced to swim [251,252]. Rats tested in the water previously swum in by another rat were significantly less immobile than rats tested in fresh water. It also confirms that alarm chemosignals affect animal behavior [253].

In addition to odors, animals are able to receive vocalizations emitted by conspecifics. Rats are able to emit two types of ultrasonic signals: vocalization at a frequency of 22 kHz (alarm signals) in aversive and dangerous situations and vocalization at a frequency of 50 kHz (appetitive) in appetitive situations [254]. Rats emit 22 kHz vocalizations in such situations as predator exposure and fighting. This type of vocalization reflects anxiety and fear and serves as alarm calls to warn conspecifics about external danger. 50 kHz vocalizations are typical for such situations as rough-and-tumble play and mating. This type of vocalization reflects the state of joy and happiness [255,256]. It is believed that 22-kHz vocalizations express anxiety in a stressful situation [257,258], and it is confirmed by the fact that rats exposed to 22-kHz ultrasonic vocalizations playback are characterized by anxiety-like behavior [259]. Predator odor can be used as a stress factor in CUMS. It is well known that rats exposed to predator odors emit 22-kHz calls, which are perceived by conspecifics as alarm signals [260]. Restraint stress which is also often used as a stress factor in the CUMS protocol, induces the emission of 22-kHz ultrasonic vocalizations in rats [261]. Social defeat stress is also often used as an animal model of depression or as one of the stressors in the CUMS protocol. The level of 22 kHz ultrasonic vocalizations increases in subordinates in the social defeat [262]. It was shown that during CUMS, the number of aversive vocalizations increases, and the number of appetitive vocalizations decreases. For example, CUMS leads to decreased levels of 50 kHz ultrasonic vocalizations and increased levels of 22 kHz ultrasonic vocalizations in rats [263,264].

Before exposing animals to CUMS they are usually divided into several groups. There is always a group of animals that is exposed to stressful factors and a control group in which animals do not experience any stressful events. Animals in the control group can visually observe the manipulations performed with animals in the stress group, as well as being able to receive vocalizations and smell the odors, experiencing psychological stress. Due to this reason, animals of the control group and animals of the stress group must be isolated from each other and kept in separate rooms in order to exclude the possible influence of psychological stress on control animals.

## 12. Rarely Considered Factors (Unknown Variables)

The physiological state and behavior of animals during CUMS can be significantly affected by a large number of various factors which are not controlled by the researchers. Different laboratories have their own sets of uncontrolled factors that affect animals. This leads to conditions for the poor reproducibility of the model not only by other groups but also by researchers from one particular laboratory if the uncontrolled factor disappears or, conversely, a new one appears. Poor reproducibility can be associated with many factors that researchers do not pay attention to but which have a significant effect on animals, being stressful in nature: frequency and duration of handling, the familiarity of the animal with the experimenter, injections, capture and restraint during injection, testing time, lighting mode-regular or reverse [265], light, noise, cage cleaning and in-house transport [266], marking/identification of animals, diet, neighbors, humidity, site of testing, the order of testing, time of day, season [267]. There are different factors of different natures, but most importantly, they are often present, regularly repeated, and unavoidable [268].

Routine laboratory procedures are stressful for animals [269,270]. Entering the animal housing room, even without handling the animals, increases the heart rate and body temperature in mice [271]. Moving the cages and opening the lids by familiar staff are stressful for rodents [272]. Transferring mice during cage change leads to a significant increase in serum corticosterone levels and influences anxiety-like behavior [273]. Familiarity of the test animals with their experimenters is also important and can influence animal behavior [274]. The sex of the experimenter and his odor can also play an important role. In particular, male experimenters and male odors produce a stronger stressful effect, causing an increase in corticosterone levels in animals [275].

Ambient noise is one of the most common uncontrolled factors. Most researchers consider their animal facilities to be quiet. However, when they start to think about this issue, they immediately find several sources of noise that can affect the physiological state of animals [276,277]. The sources of noise in animal facilities are mainly associated with human activities, and noise appears during working hours and not on weekends. It cannot be ruled out that the physiological state and behavior of animals may significantly differ in the morning on Monday (after a rather quiet weekend) and at the end of the working day on Friday (after constant noise during the working week) [278]. Noises associated with the construction/repair work in the animal facilities or in close proximity to it most clearly demonstrate the possible influence of noise on the animal’s state. During construction works, noise can lead to a twofold increase in the level of ACTH, corticosterone, and aldosterone in animals [279], as well as provoke a decrease in food intake and retardation in body weight gain [280].

Interestingly, even the physical context of stress exposure also plays an important role. The chronic homotypic restraint stress leads to HPAA habituation, which is expressed in a decrease in the level of ACTH and corticosterone in rat plasma. This process occurs regardless of the scent in the animal housing room (no scent, banana or peppermint). If the restraint stress is carried out in a room with an unfamiliar scent (change in the context), then the HPAA dishabituation occurs [281].

There are a huge number of other parameters uncontrolled or unaccounted for by the experimenter, such as type of food, water quality, the microbiological status of the animals, different levels of veterinary care, the design features of the cage (height, transparency of the walls), humidity, the location of the cages on the shelves and the amount of light that falls on them, the number of animals in the cage, frequency of animal-human interactions, light intensity, airflow from the air conditioner, the operation of the ventilation system, the season in which the experiment is conducted, the time of day of the experiment, the order of testing of animals from the same cage, the quality of bedding material, cage changing practices, etc. Obviously, such factors can be stressful for animals and sometimes even more significant in intensity than stressful factors used in CUMS. Each laboratory has its own unique set of uncontrolled factors, which makes the reproduction of any stress protocol virtually impossible.

The incompleteness of the information presented in the article makes the reproduction of the experiment difficult for other researchers. It is strongly recommended to indicate the age of the animals, housing conditions in animal rooms (temperature, humidity, ventilation, lighting, noise), housing conditions in cages (cage size, type of bedding, number of animals, frequency of cage change, handling), type of chow and water, time and details of intervention within the experiment, etc. [282,283].

## 13. Conclusions

The growing number of people suffering from depressive disorder, the lack of understanding of the etiological and pathophysiological mechanisms of the development of this mental disorder, as well as the low therapeutic efficacy of modern antidepressants indicate an urgent need for further fundamental and applied research in this field of neuroscience. Despite a number of models and tests to evaluate depressive-like behavior [284], they all have certain limitations. The CUMS model of depression is one of the best attempts to simulate the human state in animals. However, the reproducibility and reliability of the model are questionable. In this review, we tried to show that completely different factors can be the source of poor reproducibility.

The variability of individual behavioral, biochemical, and physiological reactions may be due to various factors [285], such as the stressor type (psychogenic or systemic) and characteristics of the stressor (controllability, predictability, duration, and frequency of exposure, intensity), organismic variables (species, strain, age, gender) and the ability of the organism to habituate to stress, housing, and handling.

It seems that the problem of poor reproducibility can be solved by standardization. However, it is not possible to obtain exactly the same results in different laboratories, even in the case of using animals of the same strain, assessing the same behavioral parameters according to standardized protocols, and using identical equipment [286,287]. At the same time, it is obvious that there are a large number of uncontrolled factors that researchers do not pay attention to, but the effect of such factors on the animal’s state can be more significant than the effect of stress factors included in the CUMS protocol.

We can offer the following main recommendations aimed at improving the reproducibility and etiological validity of the CUMS model:

(1) In order to determine the sucrose preference more accurately, it is recommended to measure consumption during the dark phase of the diurnal cycle (the active period in rodents), which usually lasts 12 h. It should be taken into account that the main consumption of sucrose solution by animals occurs in the dark phase.
(2) Remember that stress exposure during the inactive phase for the animal leads to chronic sleep deprivation, which itself is an additional stressful factor
(3) If injections of drugs (e.g., antidepressants) are supposed in the experimental protocol, an intact group of animals should be included in the experiment (group without any injection). If possible, avoid using stressors that can cause physical pain.
(4) Pay attention to the number of animals in the cage, their hierarchical status, the type of social stress, and its duration because social stress has a significant influence on the animals.
(5) Pay great attention to the housing and handling conditions. It affects the degree of familiarity of the animals with the experimenter.
(6) If possible, avoid using the same stressor several times during the CUMS. Strive for the “true unpredictability” of the model.
(7) Pay attention to the age of the animals and indicate it as the number of postnatal days. Animals of different ages have different susceptibilities to stress.
(8) It is necessary to increase the representation of females in a CUMS model of depression.
(9) The use of psychogenic stressors is preferable, while the use of metabolic stressors should be avoided.
(10) Control animals and animals of the stress group should be kept in separate isolated rooms to exclude the stressful effects of olfactory, visual, and auditory stimuli on animals.
(11) Try to control as many factors as possible and indicate this in the “materials and methods” section.

In conclusion, we would like to note that the presence of shortcomings in the model does not imply its rejection but only says that we need to address the controversial issues, explain the phenomenon of poor reproducibility and identify the reasons underlying it to improve this model. This signifies that we must continuously improve our technologies, methods, models, and tools in neurobiology.

## Figures and Tables

**Table 1:** Possible sources of poor reproducibility.

Factor	Comment
sucrose solution	determination of sucrose concentration threshold to select the optimal concentration
water and food deprivation	effects on the metabolic state of the animal
stress susceptibility	stress-susceptible and stress-resilient animals
time of day	stressors are often used during light phase, which causes chronic sleep deprivation which affects the physiological state of animals
painful stressors	injections, immobilization, and other painful stressors which affects the physiological state of animals
social stress	behavioral effects of isolation, overcrowding, and hierarchical rank; different types and combinations of social stress in different laboratories
strain	different susceptibility to stressors
supplier	laboratory animal supplier and housing conditions; different susceptibility to stressors
handling	handling duration and familiarity with the experimenter
habituation	effects of repeated exposure to the same stressor even in an unpredictable manner
age	different susceptibility to stressors
sex	often males; different susceptibility to stressors
type of stressful factors	the combination of stressors of various modality, the order, and duration of presentation of each stressor
stress transmission	effects of visual, auditory, and olfactory sensations on animals
unaccounted factors	a large number of uncontrollable factors affecting the physiological state of animals

## Data Availability

Not applicable.
